# Influence of Pore Size Variation on Thermal Conductivity of Open-Porous Foams

**DOI:** 10.3390/ma12122017

**Published:** 2019-06-24

**Authors:** Jakub Skibinski, Karol Cwieka, Samih Haj Ibrahim, Tomasz Wejrzanowski

**Affiliations:** Faculty of Materials Science and Engineering, Warsaw University of Technology, 02507 Warsaw, Poland; karol.cwieka@pw.edu.pl (K.C.); samih@vp.pl (S.H.I.); tomasz.wejrzanowski@pw.edu.pl (T.W.)

**Keywords:** thermal conductivity, open cell foams, finite element method, microstructure

## Abstract

This study addresses the influence of pore size variation on the effective thermal conductivity of open-cell foam structures. Numerical design procedure which renders it possible to control chosen structural parameters has been developed based on characterization of commercially available open-cell copper foams. Open-porous materials with various pore size distribution were numerically designed using the Laguerre–Voronoi Tessellations procedure. Heat transfer through an isolated structure was simulated with the finite element method. The results reveal that thermal conductivity is strongly related to porosity, which is in agreement with the literature. The influence of pore size distribution has also been observed and compared with analytical formulas proposed in the literature.

## 1. Introduction

Thermal conductivity is an essential factor in designing open-porous materials applied in heat transfer devices such as cross-flow heat exchangers, catalytic converters, electrodes of high-temperature fuel cells or solar collectors [1,2,3]. In recent years, high-porosity materials have been found attractive due to their low density and high specific strength, rendering it possible to enhance the thermal performance of devices with the simultaneous reduction of their weight and size. Additional advantage can be observed in terms of permeability, especially compared to packed bed, frequently utilized in catalytic converters and thermal energy storage devices, where it incurs a large pressure drop throughflow.

The most commonly used methods for manufacturing of commercial foam materials are: 3D printing, method based on chemical reactions with cross-linking [4], polymeric sponge method [5], directional solidification of metal from an overheated liquid state in overpressure and high vacuum [6], and foaming by powder or gas injection [2,7,8]. The abovementioned methods render it possible to control selected structural parameters of porous materials, such as porosity, mean pore size, pore size distribution or strut shape. The microstructure of open-porous foams is complex and irregular, which rules out the possibility of applying analytical approaches for studying such materials. The rapid increase in computational power in recent years, together with development of more efficient algorithms made modeling of open-porous structures possible. Many geometrical models have been proposed in the literature, representing crucial features of real porous structures. Despite recognized drawbacks and limitations, models representing the structure by ordered patterns based on regular polyhedrons (usually tetracaidecahedrons) are frequently used in studies on the properties of open-porous materials [9,10,11,12]. It turns out that, despite their random structure, a set of macroscopic properties of porous structures can be modeled with sufficient accuracy with the use of regular geometry. Experimental observations, however, clearly indicate the distribution of pore size [13] and sometimes anisotropy in real materials, which makes such simplified models insufficiently accurate. In order to increase consistency of models with real materials, other approaches for simulating properties of porous materials have been developed, namely tessellation methods and methods based on computed tomography. Each of the abovementioned approaches is based on computer modeling. In tessellation methods, the volume of the representative cell of the material is divided to smaller polyhedral volumes, taking into account predefined size and distribution. Different variants of Voronoi tessellations are successfully applied to represent random nature of foam materials [14,15], most commonly Laguerre–Voronoi tessellations (LVT) [16,17,18,19]. This algorithm, applied also by the authors of this study, enables the development of models of foam materials with controlled pore size distribution. In addition, 2D/3D reconstruction methods [20,21,22,23,24] have drawn attention of scientist in recent years, where the model of geometry is developed on the basis of reconstruction and processing of tomographic images and subsequently used in dedicated software for simulations. The geometry obtained in this manner is a direct projection of the materials structure, however parameterization of such structures or determination of general correlations is difficult. 

Quantitative relationships between microstructural parameters and properties of porous materials are the main goal in most of the studies applying models of porous structures found in the literature [25,26,27,28,29,30,31,32,33,34]. Heat transfer in foam materials is also a subject described in scientific research [35,36,37,38,39,40]. A wide literature review in this area has been performed by Zhao [41]. Thermal conductivity of porous materials has been reported to be strongly influenced by porosity. The influence of mean pore size (expressed in pores per inch—ppi) on heat transfer properties of porous materials is undertaken in research performed by Bagci [42]. Also the influence of strut shape on thermal properties of porous materials has been studied [43]. A broad study regarding the influence of microstructure on conductive heat transfer in open-cell metal foams has been performed by Coquard et al, [44,45,46], Baillis et al. [47] and Randrianalisoa et al. [48]. The described studies indicate the influence of microstructure on the thermal conductivity of open-cell foams, however most approximations and theoretical formulas only indicate porosity as the main factor determining heat transfer properties. We agree that this is the main factor influencing such properties, however other microstructural parameters also influence the heat transfer phenomena and cannot be omitted. Our previous study exhibited the influence of pore size distribution on the permeability of open-porous foams [49].

The aim of this study is to explore the influence of pore size variation on effective thermal conductivity of open-cell foams. Commercial copper foams have been characterized using computer tomography as reference structures. Based on the results obtained, a set of representative structures with various pore size distribution has been designed with use of Laguerre–Voronoi tessellations. The finite element method has been employed in order to simulate heat transfer and to calculate effective thermal conductivity of the designed materials. Correlation between effective thermal conductivity and pore size distribution has been determined. 

## 2. Materials and Methods 

Numerical models of complex porous geometries were designed by using Laguerre–Voronoi tessellations algorithm. LVT enables the generation of open-cell porous structures with predefined porosity, mean pore size and pore size variation. Self-developed code with implemented LVT algorithm firstly generates a set of spheres with predefined size distribution in a finite bounding box and afterwards packs them with the indicated degree of overlap through iterative downsizing of bounding box dimensions. In the next step, dilatation of spheres is performed, and a polycrystalline-like structure is created. Subsequently, the triple-edge model is generated by removing all volumes. To create the porous open-cell structure, cylinders with constant diameter are generated along the cell edges and spheres with diameters equal to those of the cylinders are generated in the cell vertices to assure dimensional conformity along the edges. More details about the modeling procedure can be found in [18]. 

A set of 15 structures with a coefficient of pore volume variation CV(V) ranging from 0.45 to 2.0 was developed. Coefficient of pore volume variation was set using a designed distribution of sphere diameters in the first step of the LVT procedure described above. The value of CV(V) for each structure was calculated as CV(V) for LVT cells, a ratio of standard deviation of pore volumes and mean pore volume. In Table 1 mean pore volume E(V), standard deviation of pore volumes SD(V) and coefficient of variation CV(V) values are presented. As can be noticed, the transition from LVT sphere distribution to pore volume distribution was correct, E(V) value was constant for all model structures. Hence, it is possible to determine the influence of only CV(V) on heat transfer.

Each structure was limited by a bounding box of cubic shape sized 35 × 35 × 35 mm and consisted of 200 pores. According to the literature data [20,50] this number of pores is expected to be sufficiently high to study their effect on the properties of the structures in question. Four strut diameters were selected: 0.5, 1.0, 1.5 and 2.0 mm, which resulted in porosity ranging from 97.9% to 73.8%. Simplification regarding the strut shape was applied in the model, and cylindrical struts were used, in order to reduce computational power requirements. Authors assumed that the tendency of calculated parameters would remain the same if the mean strut diameter (equivalent) was retained along with consistency of the porosity of the materials. Porosity of the material was aimed to remain constant for each set of strut diameters, although CV(V) values varied. However, due to the random nature of the LVT algorithm, small variations of porosity were obtained. Optimal discretization quality, providing numerical convergence and accurate results of heat transfer simulations, was achieved with finite element meshes consisting of above 2 million elements (finite element mesh density dependent on the strut thickness).

**Figure 1:**
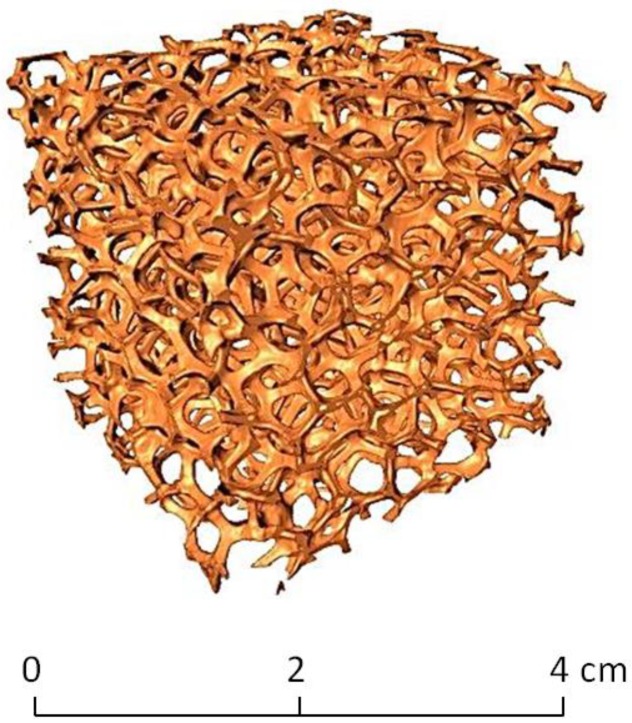
Reconstruction of tomography results of open-porous copper foam.

Commercial copper foams for heat transfer applications were used as reference for the design of virtual models. Foams with pore size of 10 pores per inch were studied with the use of SkyScan X-Radia XCT-400 in order to determine their structural parameters. Porosity, mean pore diameter and mean strut diameter were determined for validation of the virtual models generated by the design procedure. Three-dimensional image acquisition was performed under an acceleration voltage of 150kV and current of 50A, which ensured a voxel resolution of 41.49 microns. A three-dimensional reconstruction of the sample was generated by collecting a series of absorption radiographs of the cellular material. A total of 900 projections of the 2D radiography images were reconstructed using XCT XRadia reconstruction software. This process resulted in a set of 256 level greyscale bitmap-format tomograms. In order to recover the actual geometry of the specimen, this dataset had to be filtered and binarized, i.e., each pixel had to be prescribed with a value 0 or 1 (black or white) denoting void or solid phase. Both processes were performed using SkyScan CTAn software. A more detailed description of the applied methodology can be found in [51]. The segmentation method applied was the Otsu method [52]. Verification of the results was performed with a selection and tomography of a smaller sample in order to obtain a pixel size of ~23 microns, which allowed for the selection of proper segmentation and the selection of threshold levels. An example of the studied commercial material is shown in Figure 1. 

Additional SEM analysis was performed in order to assess roughness and to confirm that there are no discrepancies of the results regarding roughness of a smaller size than the scan resolution. The parameters determined by the analysis are listed in Table 2. As can be noticed, commercial foam materials with 10 ppi have similar porous structure parameters as numerical models with CV(V) equal from 0.45 to 0.48. This fact indicates that the presented methodology renders it possible to generate representative models. Additionally, numerical models with higher coefficients of pore volume variation will be studied, up to CV(V) = 2.0. In this way it will be possible to determine the role of microstructure homogeneity in the heat transfer process.

Finite element method implemented in ANSYS Mechanical software was applied to calculate the thermal properties of open- porous materials. Isolated materials with a range of CV(V) were subjected to temperature difference of 27 K in a steady state. A discrete model was developed with the use of second order tetragonal elements. In order to determine the thermal conductivity of the considered materials, Fourier law was employed:

(1) q= −k grad T=λ ∇ T

where *q* is the heat flux W/m^2^, *k* is thermal conductivity W/(mK) and *T* is temperature.

**Figure 2:**
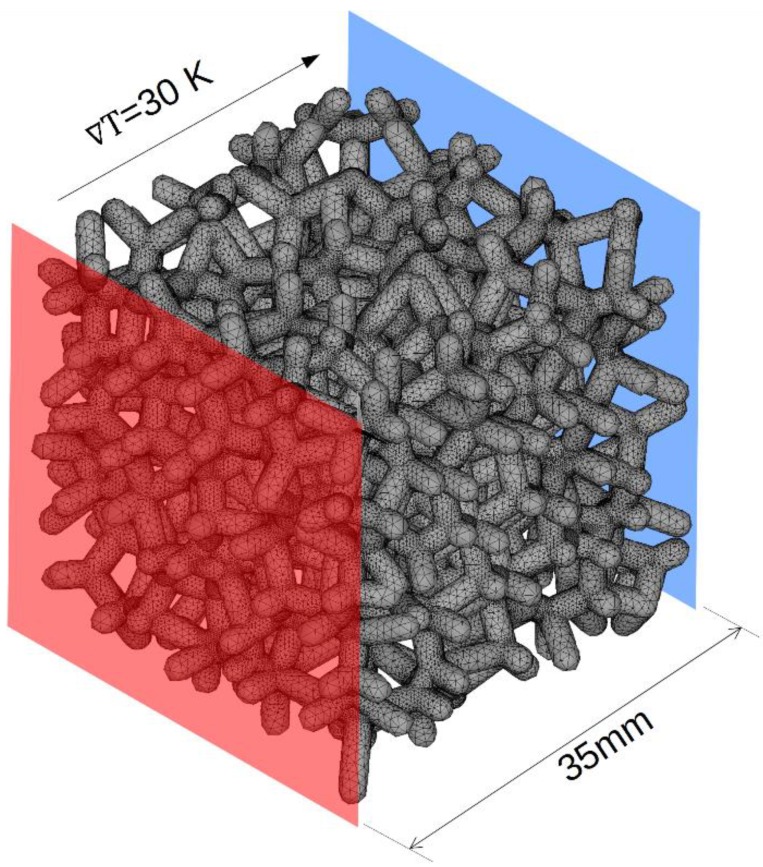
Boundary conditions for thermal conductivity calculations.

For numerical analysis, the following boundary conditions were assumed: bulk thermal conductivity 400 W/(mK), and temperature gradient ∇ T=30 K. Boundary conditions determining the heat transfer in the studied case are illustrated in Figure 2.

The aim of this study was to explore and indicate the influence of the microstructure of copper foam on the thermal conductivity of the solid fraction, isolated from the porous/fluid phase. It is well known that the porous phase accounts for the thermal conductivity of the whole material, including radiative heat transfer effect. However, the reference materials considered here are applied as cross flow heat exchangers, therefore, conditions for the fluid phase varies in terms of fluid flow velocity and temperature. A separate study is being performed in order to determine temperature transfer efficiency dependence on the structural parameters of such foams. Within the performed research, other aspects were considered in order to underline the heat transfer through the solid phase, which appeared to be more complex than one could expect from analytical approaches based solely on solid and porous fractions.

Analytical models were employed for comparison with the modeling results. A very common model applied for estimating thermal conductivity [41] is derived from the rule of mixtures:

*k_eff_* = *εk_f_* + (1 − *ε*)*k_s_* (2)

where *k_f_* is thermal conductivity of fluid phase, *k_s_* is the thermal conductivity of solid phase and *ε* is the porosity.

Such a model, however, gives a rough overestimation of the thermal conductivity because it does not account for the microstructure of the material. Another model taken into consideration was proposed in [17,40,53]:

(3) $$\frac{k_{eff}}{k_{s}}=\frac{1}{\sqrt{2}}{\{\frac{4\lambda }{2e^{2}+\pi \lambda \left(1-e\right)}+\frac{3e-2\lambda }{e^{2}}+\frac{{\left(\sqrt{2}-2e\right)}^{2}}{2\pi \lambda^{2}(1-2e\sqrt{2}}\}}^{-1}$$

where *e* is a coefficient accounting for cell geometry and:

(4) $$\lambda =\;\sqrt{\frac{\sqrt{2}\left(2-\left(\frac{5}{8}\right)e^{3}\sqrt{e}-2\varepsilon \right)}{\pi \left(3-4e\sqrt{2}-e\right)}}$$

The third model chosen for comparison was developed by Bauer [54]. The equation applied within his study is:

(5) $$(k_{eff}-k_{f})/(k_{s}-k_{f}){\left(k_{s}/k_{eff}\right)}^{1/n}=\left(1-\varepsilon \right).\;$$

where *n* is a pore shape factor determined from experimental results.

## 3. Results and Discussion

**Figure 3:**
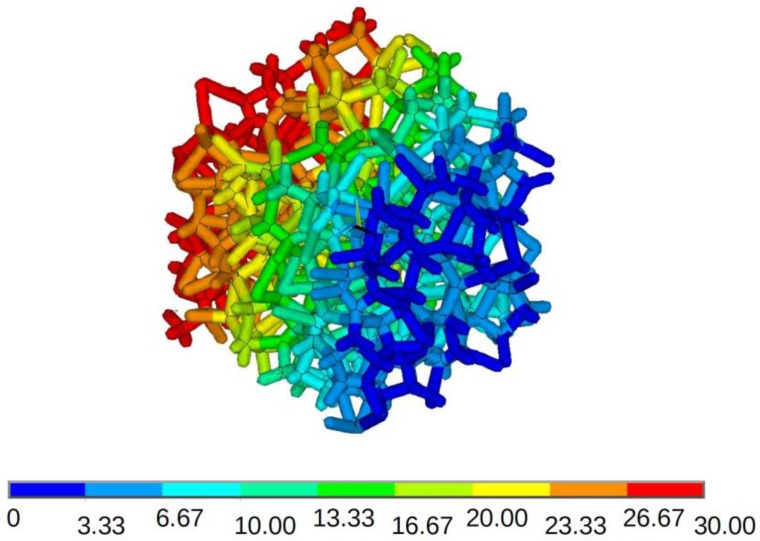
Temperature distribution in the designed structure with CV(V) = 0.47 during heat transfer simulations.

The goal of the performed analysis was to study the influence of pore size distribution on the heat transfer properties through the solid phase of open-porosity structures. Heat transfer through the studied structures, resulting from determined temperature difference, was simulated and thermal conductivity was calculated for each structure. Based on numerical simulations, heat flux for each material was determined for applied boundary conditions. An example of temperature distribution in a structure with a pore size distribution with the smallest variation (CV(V) = 0.47) during heat transfer simulations is shown in Figure 3.

Verification of the design procedure was performed through the comparison of thermal conductivity values calculated for commercial copper foams and the designed LVT open-porous materials with similar structural parameters (Table 3). Binary images obtained from computed tomography were converted into a virtual model. Subsequently, finite element calculations were performed, and thermal conductivity of commercial materials was determined. Comparison of the calculated values of thermal conductivity is shown in Table 3. Results are in satisfactory agreement, which was considered by the authors as a verification of the design procedure.

**Figure 4:**
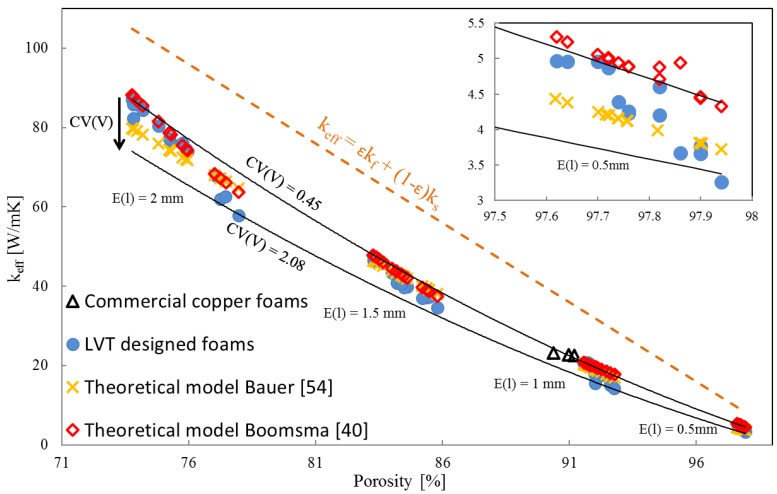
Correlation of thermal conductivity of the designed materials on porosity.

Taking into account the influence of porosity variation on the thermal conductivity of studied structures, values of effective thermal conductivity (*k_eff_*, thermal conductivity of the solid phase) were compared for four different values of mean strut diameter (E(l)): 0.5, 1, 1.5 and 2 mm for the same range of CV(V) values (Figure 4).

The influence of pore size distribution is noticeable. The anisotropy of the samples was also studied by calculating the heat transfer coefficient in three directions for each sample. The values did not differ by more than 3%, nevertheless the *k_eff_* of the designed structures presented in Figure 4 are the average values from three perpendicular directions. Based on the above chart it can be concluded that the heat flow phenomenon through the solid phase strongly depends on strut diameter and strut connections. The simple mixing rule cannot be applied to estimate *k_eff_*, because other microstructural parameters play an important role. Thermal conductivity is not solely exponentially dependent on porosity, and the increase of pore size distribution causes a significant drop of thermal conductivity value. For heat transfer devices, where higher thermal conductivity of the material is desired, open-porous materials with homogeneous pore size variation should be recommended, while for materials applied as isolators, non-homogeneous pore size distribution would be a better choice. 

It can be noted, that the rule of mixtures does not account for the microstructure of the material, hence it provides a significant overestimation of the effective thermal conductivity of open-porous foams. The theoretical model proposed by Boomsma et al. [40] gives a good estimation of effective thermal conductivity, so the coefficient of cell geometry *e* is also properly fitted. The results obtained demonstrate that the effective thermal conductivity calculated for materials with a higher coefficient of pore size variation, CV(V), is lower than the value obtained from Equation (3). In the case of inhomogeneous pore size distribution, the theoretical model [40] gives an overestimation of *k_eff_*.

## 4. Conclusions

We used Laguerre–Voronoi tessellations and the finite element method to study the influence of microstructural characteristics: porosity and pore volume distribution, on the effective thermal conductivity in open-cell porous materials. The design procedure was used to create a set of virtual structures with various coefficients of pore volume variation and structural parameters corresponding to commercial copper foams. The results obtained reveal the influence of the structural parameters of open-porous materials on their thermal conductivity. Results also indicate that there is a strong dependence between thermal conductivity and the pore volume variation coefficient. Higher thermal conductivity can be obtained for structures with homogeneous pore volume distribution. Due to the differences in porosity between designed structures, resulting from the stochastic nature of the applied algorithm, it was not possible to derive an exact relationship describing thermal conductivity as a function of CV(V), but such an influence has been exhibited. The results obtained were compared with theoretical models described in the literature. Effective thermal conductivity values of structures with inhomogeneous pore size distribution indicate, that simple theoretical models (derived from the rule of mixtures) overestimate *k_eff_* for such structures. The finding that different thermal conductivity can be obtained for open-porous materials due to the inhomogeneity in pore size distribution can be used to optimize the microstructure of foams for various relevant applications.

## Figures and Tables

**Table 1:** Values of mean pore volume (E(V)), standard deviation of pore volumes (SD(V)) and resulting coefficient of variations (CV(V)) for generated models.

**CV(V)**	0.48	0.85	1.26	1.69	2.08
**E(V)**	0.175	0.175	0.175	0.175	0.175
**SD(V)**	0.0837	0.1503	0.2206	0.2967	0.3647

**Table 2:** Microstructural parameters of designed and commercial materials. LVT: Laguerre–Voronoi tessellations.

Structure	Commercial Materials			Numerical Models		
	10 ppi No 1	10 ppi No 2	10 ppi No 3	LVT CV(V) = 0.458	LVT CV(V) = 0.478	LVT CV(V) = 0.484
Porosity (%)	90.98	90.37	91.19	91,71	91,78	91,85
Mean pore diameter (mm)	3.753	3.836	3.917	3.31	3.34	3.42
Mean strut diameter (mm)	1.144	1.192	1.137	1.00	1.00	1.00

**Table 3:** Effective thermal conductivity (*k_eff_*) of 10 ppi commercial foams and designed LVT structures.

Structure	Commercial Materials			Numerical Models		
	10 ppi No 1	10 ppi No 2	10 ppi No 3	LVT CV(V) = 0.458	LVT CV(V) = 0.478	LVT CV(V) = 0.484
*k_eff_* (W/mK)	22.71	23.20	22.54	20.52	20.44	20.38

## Abbreviations

**Symbol**	**Definition**	**Unit**
CV(V)	Coefficient of variation for pore volume distribution	-
∇ T	Temperature gradient	K
ε	Porosity	%
E(l)	Mean strut diameter	mm
SD(V)	Standard deviation of pore volumes	-
k	Thermal conductivity	W/(mK)
k_eff_	Effective thermal conductivity	W/(mK)
k_f_	Thermal conductivity of fluid phase	W/(mK)
k_s_	Thermal conductivity of solid phase	W/(mK)
LVT	Laguerre-Voronoi Tessellation	-
n	Pore shape factor	-
q	Heat flux	W/m^2^
ppi	Pores Per Inch	-
SEM	Scanning Electron Microscopy	-
